# Leakage-Proof and High-Conductivity Composite Phase Change Material Using Low-Melting-Point-Alloy-Encapsulated Copper Foam/Paraffin for Superior Thermal Homogeneity in Lithium-Ion Battery Modules

**DOI:** 10.3390/ma18194604

**Published:** 2025-10-04

**Authors:** Shengzhi He, Jiajun Zhao, Dongxu Ouyang, Mingyi Chen

**Affiliations:** 1 School of the Environment and Safety Engineering, Jiangsu University, Zhenjiang 212013, China; 2212409039@stmail.ujs.edu.cn (S.H.); zjj@stmail.ujs.edu.cn (J.Z.); 2 School of Safety Science and Engineering, Nanjing Tech University, Nanjing 210009, China

**Keywords:** lithium-ion battery, thermal management, composite phase change material, copper foam, low melting point metal alloy

## Abstract

Ensuring thermal stability is a major concern in lithium-ion battery systems. Although phase change materials (PCMs) provide a passive approach for temperature regulation, they are limited by poor heat conduction and potential leakage during phase transitions. This study develops a novel composite PCM (CPCM) using paraffin (PA) as the matrix, copper foam (CF) as a conductive skeleton (10–30 pores per inch, PPI), and a low-melting-point alloy (LMA) as an encapsulant to prevent leakage. The effects of CF pore size on thermal conductivity, impregnation ratio, and leakage resistance were systematically investigated. Results show that CPCM with 10 PPI CF achieved the highest thermal conductivity (4.42 W·m^−1^·K^−1^), while LMA encapsulation effectively eliminated leakage. The thermal management performance was evaluated on both a single 18,650 LIB cell and a 2S2P module during rate discharging at 1C, 2C, and 3C. For the module at 3C, the 10 PPI CPCM significantly lowered the maximum temperature from 75.9 °C to 44.6 °C and critically reduced the maximum temperature difference between cells from 10.2 °C to a safe level of 1.2 °C, significantly improving temperature uniformity. This work provides a high-conductivity and leakage-proof CPCM solution based on LMA-encapsulated CF/PA for enhanced thermal safety and uniformity in LIB modules.

## 1. Introduction

The growing global emphasis on environmental protection and energy sustainability is driving the development of new energy technologies as a key force in societal progress [1]. Lithium-ion batteries (LIBs) have become a primary energy storage solution, widely used in smartphones, electric vehicles, and energy storage systems thanks to their high energy density, long cycle life and rapid charging capability [2,3]. However, thermal safety remains a major bottleneck limiting their industrial development. Inadequate heat dissipation during the charge–discharge cycles of lithium-ion batteries can lead to localized heat accumulation. This temperature rise, in turn, accelerates the degradation of battery components and compromises their operational reliability [4,5]. More critically, when the temperature exceeds a critical threshold, a series of exothermic chain reactions—such as the breakdown of the solid electrolyte interphase (SEI) and separator meltdown—can be initiated, ultimately precipitating thermal runaway. This process is frequently characterized by the emission of toxic gases, open flame, or explosive events, presenting serious hazards [6,7]. This underscores the urgent need for the development of efficient, lightweight, and reliable battery thermal management systems (BTMS), which are critical for the large-scale deployment of LIB technology.

Currently, mainstream BTMS primarily employ three cooling strategies: (i) air cooling [8], (ii) liquid cooling [9,10], (iii) phase change material (PCM) cooling [11,12]. Among them, air cooling features a simple structure and is lightweight, but its application under high-rate conditions is constrained by low thermal conductivity, which compromises temperature uniformity [13]. Liquid cooling provides high heat dissipation efficiency but is hindered by structural complexity, stringent sealing requirements, and high costs [14]. Beyond these basic solutions, researchers are actively exploring more efficient hybrid thermal management systems, such as systems that integrate liquid cooling channels and phase change material [15] or integrated heat pipe technology [16]. Although these hybrid systems are designed to synergistically leverage the advantages of different cooling methods, their complexity, cost, and reliability remain critical factors that must be carefully balanced in practical applications. In contrast, passive thermal management systems based on PCM continue to attract research interest due to their inherent advantages, including simple structure, zero additional energy consumption, and stable temperature regulation. These systems show great potential, especially in applications that prioritize lightweight design, low cost, and moderate power requirements [17]. Phase change materials (PCMs) are substances that absorb, store, and release large amounts of latent heat during an isothermal phase transition process (e.g., solid to liquid). The thermal management mechanism of PCMs relies on latent heat absorption during phase transition, which allows them to store substantial thermal energy and release it isothermally. Furthermore, their compact form factor affords considerable flexibility for incorporation into diverse system designs [18]. The work of Lamrani et al. [19] employed a simplified model to validate the applicability of PCM in battery packs, demonstrating a reduction in the maximum battery temperature of approximately 3 °C. Yan et al. [20] compared natural convection with PCM systems and confirmed that PCM enhances thermal homogeneity and effectively mitigates temperature rise. Ling et al. [21] emphasized the phase change temperature as a critical parameter for selecting PCMs. They proposed that composite materials with a phase change temperature falling within the range of 40–45 °C should be selected, since deviations from this optimal window can adversely affect the efficacy of battery cooling. Phase change materials are mainly classified into three categories: organic, inorganic and eutectic [22]. In the field of inorganic phase change materials, for instance, Hassan et al. [23] developed a binary lithium salt mixture of LiCl-LiOH. The research found that samples with a composition of 32 mol% LiCl-68 mol% LiOH melted within the range of 269 to 292 °C, with a melting heat of 379 J/g. And they still showed good thermal reliability after 30 cold and hot cycles. Among organic phase change materials, paraffin-based PCMs have been widely applied in battery thermal management due to their excellent heat storage capacity and phase change performance, with approximately 90% of related research using paraffin as the core material [24,25]. At present, there is relatively little research on eutectic phase change materials [26]. However, the inherently low thermal conductivity of pure PCM can lead to uneven temperature distribution within battery modules, and there is a susceptible to leakage during the phase change process. These shortcomings significantly limit its practical effectiveness [27]. To overcome the limitations of pure PCM, researchers have developed composite phase change materials (CPCM) to enhance their overall performance. Composite phase change materials (CPCMs) are advanced PCMs that incorporate functional additives (e.g., highly conductive fillers) into a PCM matrix to overcome inherent limitations such as low thermal conductivity and leakage [24]. Common additives include metal foams [28], carbon foams [29], expanded graphite (EG) [30], carbon nanotubes [31], graphene [32], nano silica [33], and low-melting-point metal alloy (LMA) [34]. For instance, R. Bharathiraja et al. [35] doped graphene nanosheets with nano-SiO_2_ into paraffin. The thermal conductivity of the HYB4 composite sample prepared (98% paraffin +1% graphene +1% nano-SiO_2_) was approximately 50% higher than that of pure paraffin. Mills et al. [36] significantly enhanced the thermal conductivity of a material by loading paraffin onto a porous graphite matrix and successfully applied it to the passive thermal management system of lithium-ion battery packs, effectively suppressing the temperature rise in the battery. Therefore, by optimizing the performance of phase change materials, their applicability in the thermal management applications of lithium-ion batteries can be enhanced.

Metal foam, serving as a representative porous scaffold with high thermal conductivity, has garnered significant interest in the fabrication of composite phase change materials (CPCM). This is attributed to its exceptionally high open porosity (85–98%), extensive surface area, and robust mechanical stability. The mechanism of action is primarily reflected in two aspects. First, the continuous skeletal framework of the metal foam establishes an efficient pathway for heat conduction, which markedly minimizes interfacial thermal resistance and consequently boosts the bulk thermal conductivity of the CPCM. Second, its porous structure effectively restricts the flow of molten PCM, suppresses internal natural convection, and prevents local heat accumulation, contributing to a more uniform temperature distribution across the battery module [17]. Foam copper, with its higher thermal conductivity (387 W·m^−1^·K^−1^), high porosity, and structural stability, makes it an ideal support for enhancing heat transfer in CPCM. Xiao et al. [37] employed the vacuum-assisted method to impregnate paraffin (PA) into copper and nickel foams with varying porosity and pore size, confirming that metal foam can significantly enhance the effective thermal conductivity of CPCM. Huang et al. [38] prepared a myristic metal foam/alcohol (MA) CPCM using the vacuum fusion osmosis method, and found that, compared to pure MA, the thermal conductivity was enhanced by a factor of 1.80 for MA/nickel foam (40 PPI) and 7.51 for MA/C F (40 PPI). Jin et al. [39] investigated the effects of 15 PPI, 30 PPI, and 50 PPI CF on the melting heat transfer of paraffin. The results showed that under a wall superheat of 20 °C, the melting rates of 30 and 50 PPI CF were similar and significantly higher than that of 15 PPI. At 30 °C, the 30 PPI configuration was found to be optimal, with a melting rate much faster than that of the 50 PPI configuration. Liu et al. [40] designed a hybrid thermal management system integrating a phase change/copper foam (CF) composite with a helical liquid channel. Numerical simulations demonstrated that this system achieved a temperature reduction over 30 K greater than that of natural convection. The best performance was observed at a porosity of 92%, with further improvements as porosity increased. Hussain et al. [41] compared the performance of nickel foam/paraffin and copper foam/paraffin in battery applications and found that, under a 2C discharge rate, the copper foam/paraffin composite reduced the battery surface temperature by 34% compared to natural convection and by 25% compared to pure PCM, demonstrating the superior efficacy of copper foam in augmenting the thermal management capabilities of PCM.

Additionally, liquid metals (LM), especially LMA, have shown great potential as emerging functional materials in advanced thermal management due to their exceptionally high thermal conductivity and volumetric latent heat density [42]. LMA-based microcapsules, prepared by Wang et al. [43] using the liquid phase microencapsulation method, exhibited excellent latent heat storage capacity and thermal conductivity. Zhao et al. [44] explored a low-melting-point Bi-Pb-Cs-Cd alloy as a PCM, whose thermal conductivity (~125.22 W·m^−1^·K^−1^) and volumetric latent heat (approximately 365.5 MJ/m^3^) were significantly higher than those of conventional organic PCMs. Numerical simulations conducted by Tianrui et al. [45] demonstrated that a copper foam/47 alloy composite exhibits superior thermal management performance compared to the copper foam/n-triacontane system.

The innovation of this study lies in integrating a low-melting-point alloy (LMA) as a sealing layer onto a paraffin/foam copper (PA/CF) composite phase change material (CPCM). Although previous research mainly focused on using metal foams alone to enhance thermal conductivity, the leakage problem remains a challenge. This scheme adopts a stepwise enhancement strategy: using paraffin with high latent heat as the heat storage substrate. By taking advantage of the porous structure and high thermal conductivity of copper foam, the thermal conductivity performance is improved. Ultimately, LMA was innovatively introduced as a sealing layer to suppress leakage. The dense metal layer formed by its solidification can achieve physical encapsulation, thereby jointly solving the problems of low thermal conductivity and easy leakage of paraffin-based PCM. Furthermore, we provide a systematic investigation into the effect of copper foam pore size (10, 20, and 30 PPI) on key properties such as impregnation ratio and thermal conductivity, and a comprehensive evaluation is carried out on the actual temperature control effect in lithium-ion battery cells and modules under various discharge rate conditions (1C, 2C, 3C). The research outcomes are expected to provide experimental evidence for elucidating the synergistic heat transfer mechanism between porous metal frameworks and high thermal conductivity encapsulation materials, and to offer valuable theoretical support and technical guidance for the optimal design of efficient and reliable lithium-ion battery thermal management systems.

## 2. Experiment

### 2.1. Materials

The CPCM was prepared using the following raw materials: paraffin (PA) was purchased from Shengbang Polymer Materials Co., Ltd. (Dongguan, China), and was an industrial grade product with a purity of ≥99%, a phase change temperature of 42 °C, and a latent heat of 220 J/g. A low-melting-point alloy (LMA) was obtained from Dongguan Dingguan Metal Technology Co., Ltd. (Dongguan, China), a Sn-Bi-Pb-Cd alloy with a mass ratio of 10-20% Sn, 40-50% Bi, 20-28% Pb, and 5-10% Cd, a purity of 99.99%, and a melting point of 70 °C. Copper foam (CF) was sourced from Kunshan Teng’erhui Electronic Technology Co., Ltd. (Suzhou, China), with a purity of ≥99.9%, a uniform size of 52 mm × 52 mm × 65 mm (consistent across all pore size groups: 10 PPI, 20 PPI, and 30 PPI), and a porosity of 98%.

### 2.2. Preparation Method

The preparation process was carried out as follows: paraffin was used as the phase change material, while copper foam served as both the porous thermal conductive carrier and the supporting material. A high thermal conductivity CPCM was fabricated by combining PA and CF using the impregnation method, followed by encapsulation with LMA.

**Figure 1:**
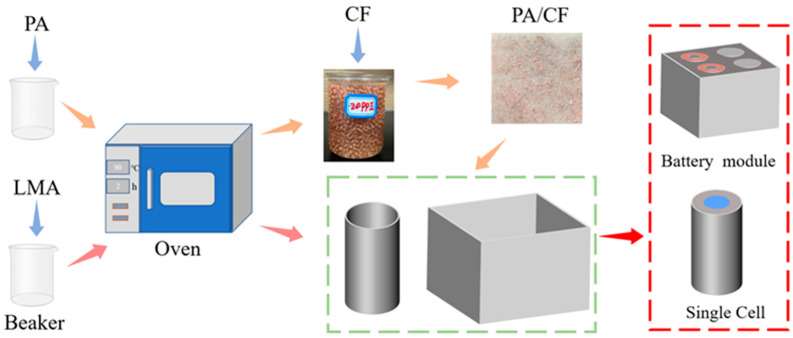
The preparation process of LMA-encapsulated copper foam/paraffin CPCM and its application principle in the thermal management of lithium batteries. Notes: The procedure involves (i) impregnating dried copper foam (CF) into molten paraffin (PA), (ii) cooling to form the PA/CF composite, (iii) encapsulating the composite with a low-melting-point alloy (LMA) to obtain the final leakage-proof CPCM.

First, a sufficient amount of paraffin powder was placed in a beaker and heated in an oven at 100 °C for 2 h to ensure complete melting (a larger quantity of paraffin and extended heating time were required to fully submerge the copper foam in the liquid paraffin). Subsequently, the copper foam was dried in an oven at 100 °C for 2 h and then immersed in the beaker containing the melted paraffin, ensuring it was fully enveloped. The prepared composite precursor was then transferred to an oven to allow for complete impregnation, ensuring the liquid paraffin saturated the porous network of the copper foam. Subsequently, the sample was removed from the oven and allowed to cool naturally to room temperature under ambient conditions, yielding the final paraffin/copper foam CPCM. Finally, encapsulation was performed on the prepared paraffin/copper foam using LMA. The prepared samples primarily fell into two categories: unencapsulated paraffin/copper foam composite phase change materials (P-series) and their LMA-encapsulated counterparts (F-series). Specifically, copper foams with three different pore sizes (10, 20, and 30 PPI) were combined with paraffin (PA) to prepare samples denoted as P1, P2, and P3. Subsequently, these three composites were encapsulated with LMA, resulting in the corresponding samples F1, F2, and F3. The entire preparation process was straightforward and practical, as illustrated in Figure 1.

### 2.3. Material Characterization

#### 2.3.1. Structure and Impregnation Effect

A Sony HDR-CX405 video camera was employed to capture the initial morphology exhibited by copper foam samples with different pore sizes, as well as the resulting macroscopic morphology after compounding with paraffin, in order to observe the filling state of paraffin within the framework of the copper foam. The mass of the copper foam before and after impregnation was measured using an SN-FA1204 electronic balance (accuracy: 0.1 mg). Based on these measurements, the impregnation ratio and porosity were calculated to evaluate the compatibility between paraffin and copper foam as well as the filling effect.

#### 2.3.2. Thermophysical Properties

The thermal conductivity of the composite phase change material (CPCM) was characterized with a Dra-III multifunctional rapid thermal conductivity meter (Xiangtan Xiangyi Instrument Co., Ltd., Xiangtan, China), employing the Transient Plane Source (TPS) method. This technique determines thermal conductivity by analyzing the transient temperature response of a disk-shaped sensor acting as a heat source within an infinite medium. The samples were prepared in a cuboid shape (40 mm × 40 mm × 10 mm), and their surfaces were polished to achieve a smooth finish and ensure uniform contact with the probe. Measurements of the thermal conductivity coefficient were conducted at room temperature. Before formal testing, 2–3 sets of preliminary trials were performed to stabilize the thermal conductivity. The formal test was conducted with five replicates, and their arithmetic mean was used to mitigate experimental variability. The instrument’s relative error was ≤3%, and the repeatability error was ≤3%.

A Testo 872 infrared thermal imager was employed to capture the phase transition process and the associated temperature field of the CPCM. A liquid-state copper foam sample impregnated with paraffin (at 80 °C) was placed in an environment at 25 °C. Infrared images were captured to evaluate the heat transfer capacity and dynamic thermal behavior of the resulting copper foam/paraffin composite.

The prepared CPCM was placed on filter paper and continuously heated at 65 °C for 2 h in a DHG-9070A electric thermostatic blast drying oven (Shanghai, China) to perform the leakage test. Changes in the sample morphology and liquid absorption by the filter paper were observed to evaluate the leakage performance of the CPCM.

### 2.4. Experimental Setup

**Figure 2:**
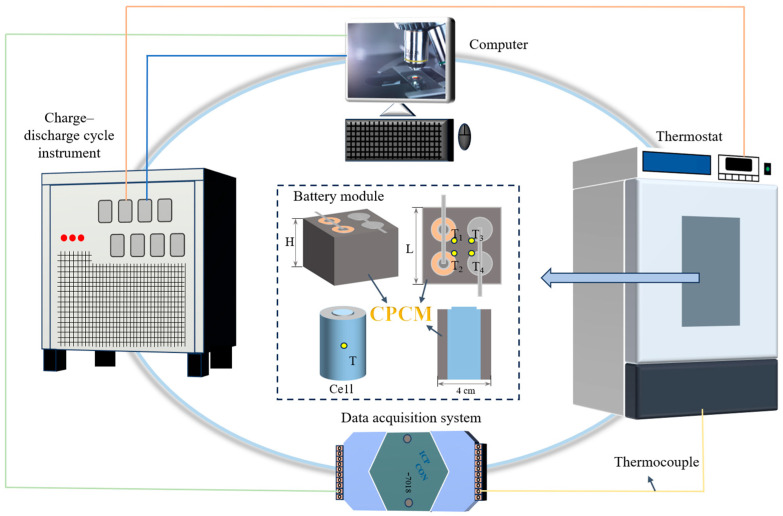
Configuration of the experimental platform.

The 18,650 lithium-ion battery (Sanyo) used in the experiment has a rated voltage of 3.7 V and a rated capacity of 3.2 Ah. After being fully charged, its voltage reaches 4.2 V. The battery size is Φ 18 mm × 65 mm, and the specified cycle life is 1000 charge and discharge cycles. The cathode material is graphite, and the cathode material is lithium nickel cobalt manganese oxide (LiNi_x_Co_y_Mn_1−x−y_O_2_). The electrolyte is composed of lithium salts, organic solvents and carbonates. The configuration of the experimental platform is shown in Figure 2. The core equipment includes the constant temperature chamber (SPX-50B, Enyi, ±0.5 °C), which is used to maintain an ambient temperature of 25 °C; the charge–discharge tester (CT-4008-10V20A-NTFA, NEWARE, ±0.1%), which is used to control the charge–discharge protocol; the T-type thermocouple (OMEG, ±0.5 °C), which is positioned at the center of the battery surface and at symmetrical locations on the module; and the data acquisition module (I-7018, ICP CON, ±0.1%), which transmits temperature data to the computer in real time. The test unit consists of either a single cell or a battery module (composed of four cells welded in a 2-series 2-parallel configuration, measuring 52 mm × 52 mm × 65 mm.), externally coated with CPCM for thermal management. The specific operating conditions of the experiment are listed in Table 1. The discharge rate is set at three levels (1C, 2C, and 3C, “C” is a relative unit representing the multiple of battery capacity), while the charge rate is maintained at 1C. The rest time after charging is set longer (10 min) than after discharging (5 min) to ensure the battery modules return to a uniform thermal baseline before each discharge cycle, enhancing the comparability of the results. The discharge capacity for each cycle was defined by the constant current discharge protocol based on the rated capacity (3.2 Ah). The actual delivered capacity under these conditions is expected to be very close to the theoretical value, as the tests were short-term and conducted at a stable temperature. The experiments were conducted repeatedly.

## 3. Results and Discussion

### 3.1. Performance Characterization of CPCM

#### 3.1.1. Structural Analysis of Copper Foam and Paraffin/Copper Foam Composite Materials

**Figure 3:**
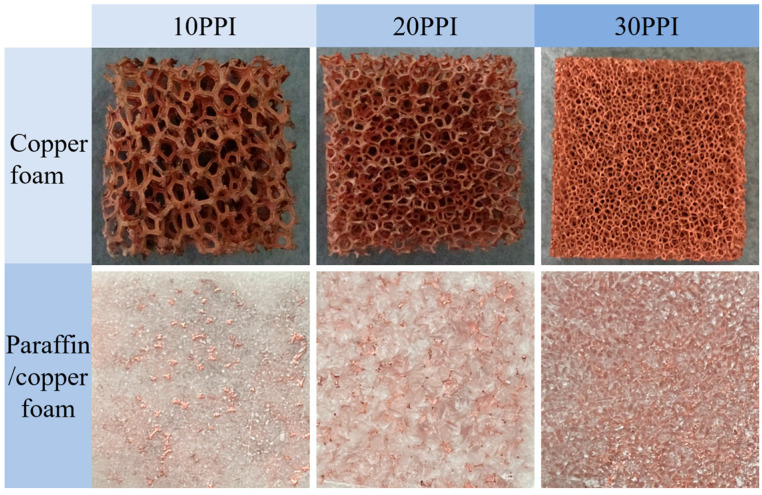
Copper foam and paraffin/copper foam CPCM samples with different pore sizes.

Figure 3 shows images of copper foam and copper foam/paraffin CPCM samples with varying pore size. The copper foam samples have pores-per-inch values of 10, 20, and 30. The sample with 30 pores per inch displays a denser pore distribution than the others. The pore size values of the copper foam with 10, 20, and 30 PPI are 2.98 mm, 1.31 mm, and 0.82 mm, respectively. The surfaces of the copper foam/paraffin CPCM with 10 and 20 PPI appear relatively rough, with more visible metal framework on the surface, mainly due to the sparse metal support structure. In contrast, the copper foam/paraffin CPCM with 30 PPI exhibits a smooth and compact surface with a darker color, which is attributed to its denser framework carrier.

#### 3.1.2. Analysis of Impregnation Effect

Equation (1) is utilized to compute the porosity of copper foam. A dimensionless parameter, as defined in Equation (2), is employed to gauge the compatibility of paraffin with copper foam exhibiting diverse pore sizes. The latent heat, a core metric for evaluating the thermal storage capacity of CPCM, is calculated using Equation (3)

(1) $$\varepsilon =1-\frac{m_{CF}}{\rho_{Sk}\cdot V_{t}}$$

(2) $$\eta =\frac{m_{actual}}{m_{theoretical}}=\frac{m_{after}-m_{before}}{\varepsilon \cdot V_{t}\cdot \rho_{CPCM}}$$

(3) $${∆H}_{CPCM}\approx {∆H}_{PA}\times \eta$$

Here, ε denotes the porosity; $\rho_{Sk}$ (g/cm^3^) represents the density of the skeleton material; $V_{t}$ (cm^3^) stands for the total volume of the CF; $m_{CF}$ (g) is the mass of the copper foam; $m_{theoretical}$ (g) refers to the theoretical mass of the CPCM; $m_{actual}$ refers to the actual mass of the CPCM; $\rho_{CPCM}$ (g/cm^3^) is the density of the solid-state CPCM; $m_{before}$ (g) is the mass of the copper foam prior to impregnation (equivalent to $m_{CF}$ in Equation (1)); $m_{after}$ (g) is the composite after the impregnation process. The impregnation ratio η (%) quantifies the efficacy of paraffin loading by comparing the actual mass of paraffin retained within the foam to the maximum theoretical uptake capacity of the porous copper skeleton. Theoretically, when all the pores inside the copper foam are fully impregnated with paraffin, the impregnation ratio reaches 100%. ${∆H}_{PA}$ is the phase change latent heat of the selected paraffin, 220 J/g, which represents the heat absorbed during its solid-to-liquid phase transition. ${∆H}_{CPCM}$ is the phase change latent heat of the composite phase change material (CPCM), i.e., the total heat absorbed or released by the material during phase transition (usually the transition between solid and liquid states). Its value is mainly determined by the latent heat of the matrix phase change material (such as PA, in this study) and its mass proportion in CPCM; since CF only acts as a thermal conductive skeleton and LMA only serves as an encapsulation layer; neither undergoes phase transition, and thus they contribute no latent heat. In the experiment, the dimensions of the copper foam were 40 mm × 40 mm × 10 mm. The relevant parameters of the copper foam/paraffin qin are listed in Table 2.

**Figure 4:**
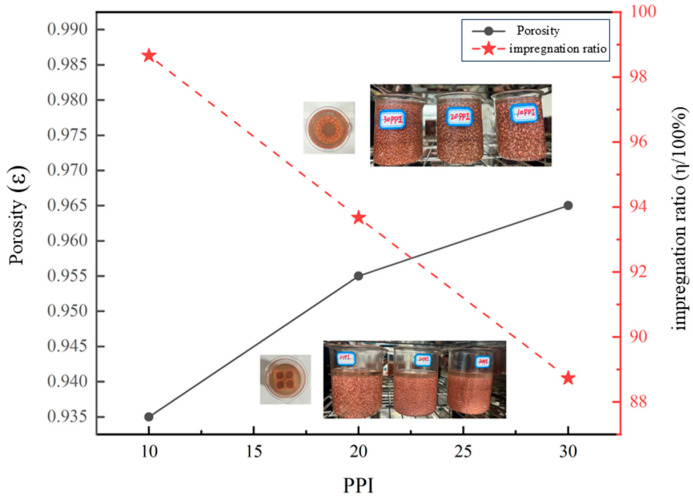
Impregnation ratio as a function of porosity for copper foams with different pore sizes.

Figure 4 shows the variation curves of impregnation ratio and porosity for copper foam/paraffin CPCM samples with three different pore sizes as a function of copper foam pore size PPI. As shown in the figure, porosity increases with increasing copper foam PPI, which is due to the fact that higher PPI values correspond to a lower amount of copper foam used. Therefore, under the same volume, porosity increases as the copper foam PPI increases. The impregnation ratio decreases with increasing PPI, as a higher PPI in the copper foam leads to a denser and more irregular pore structure. Additionally, the internal pores of the copper foam become smaller as the PPI increases. These two factors together hinder the full penetration of liquid-state paraffin into the entire structure of the copper foam. The impregnation ratio of copper foam/paraffin with different PPI values remains less than 100%, because during the solidification process of the copper foam/paraffin CPCM, the external paraffin solidifies before the internal paraffin, which then solidifies subsequently. Due to the density difference between the solid and liquid states of paraffin, the internal paraffin inevitably undergoes shrinkage. This inconsistent shrinkage leads to the formation of micro-voids within the CPCM, preventing the impregnation ratio of the PA/CF CPCM from reaching 100%. Since only PA contributes to latent heat (CF and LMA do not undergo phase transitions), ${∆H}_{CPCM}$ can be quantified using ${∆H}_{PA}$ and the η . The decrease in ${∆H}_{CPCM}$ with increasing CF PPI is due to the denser and more irregular pore structure of high-PPI CF. This trend confirms that higher PA impregnation (from lower-PPI CF) helps retain the latent heat of the CPCM, laying a foundation for its thermal management performance.

#### 3.1.3. Thermal Conductivity

**Figure 5:**
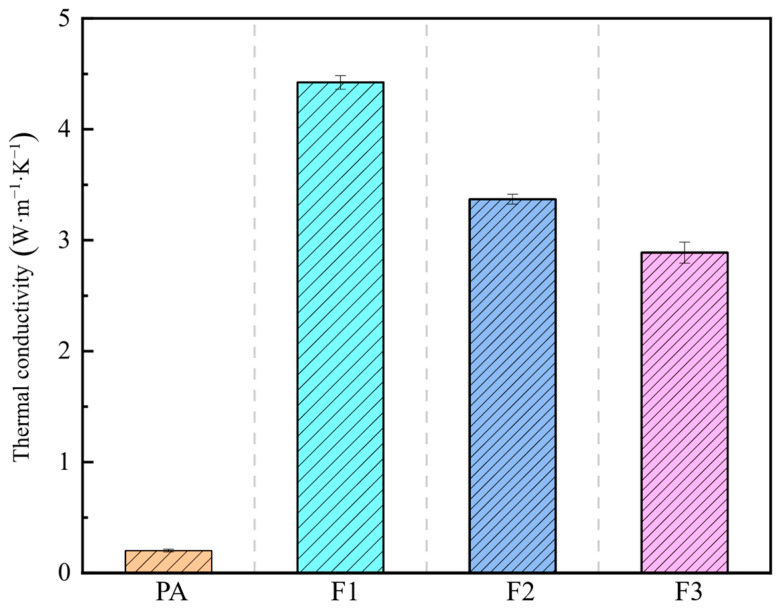
Thermal conductivity of PA and different PA/CF/LMA CPCM samples.

The thermal conductivity test results for the three PA/CF/LMA CPCM samples with different pore sizes are presented in Figure 5. The results indicate that the thermal conductivity of the CPCM decreases as the copper foam PPI increases. When the PPI of the copper foam increases from 10 to 30, the thermal conductivity coefficient drops from 4.42 W·m^−1^·K^−1^ to 2.89 W·m^−1^·K^−1^. This phenomenon can be mainly attributed to two factors: (1) Increased contact thermal resistance: As the PPI increases (i.e., the pore size decreases), the metal framework becomes finer. Although the contact area between the paraffin and the metal framework increases, the microscopic contact may be less effective than in samples with larger pore size, leading to higher contact thermal resistance. (2) Reduced metal framework proportion: As shown in Table 2, higher PPI copper foam has greater porosity, meaning that the proportion of the high thermal conductivity metal framework within the same volume of CPCM is lower. The combined effect of these two factors results in a reduction in the thermal conductivity of high PPI CPCM. Experimental findings confirm that the porous network within the copper foam, along with its high thermal conductivity metal properties, provides a synergistic enhancement that improves the thermal conductivity of the CPCM.

#### 3.1.4. Infrared Thermographic Analysis

**Figure 6:**
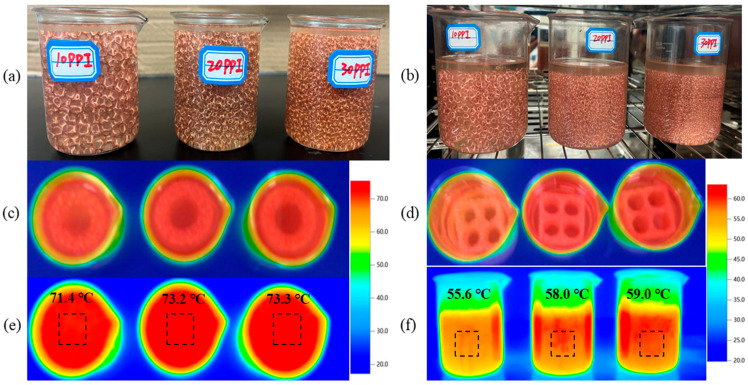
An analysis comparing optical images alongside the infrared images of three different pore sizes of CF/PA CPCM: (**a**) shows the front view of the sample applied to the battery; (**b**) shows the front view of the sample applied to the battery module; (**c**–**f**) show the top views and corresponding infrared images of the samples applied to the battery and battery module.

To visually reveal the temperature variation process of CPCM, copper foams with pores per inch of 10, 20, and 30 were placed in a vacuum drying oven and heated up to 80 °C. After the paraffin was completely infused into the pore structures of the copper foam, the three different liquid copper foam–paraffin CPCM were taken out of the oven. Transient temperature response and heat transfer performance of the copper foam/paraffin composite CPCM were then observed using an infrared thermal imaging camera. Figure 6 shows visual photos and their corresponding infrared-based thermal imagery of three PA/CF CPCM specimens (P1: 10 PPI, P2: 20 PPI, P3: 30 PPI) used in single-cell and battery-module thermal management scenarios. In the single-cell application (Figure 6a,c,e), the surface temperature of the samples increased with higher PPI values (P1: lowest at 71.4 °C; P2: 73.2 °C; P3: highest at 73.3 °C), which aligns with the thermal conductivity test results. A larger pore size (i.e., lower PPI) corresponds to thicker thermally conductive metal ligament, leading to an enhanced heat transfer rate and enhanced heat dissipation capability of the CPCM to the surrounding environment. In the application in a battery module (Figure 6b,d,f), analysis of the corresponding data reveals an analogous behavior: the surface temperature of the samples increased with increasing PPI (P1 lowest: 55.6 °C; P2: 58.0 °C; P3 highest: 59.0 °C). This suggests that copper foams with different pore sizes exhibit a consistent pattern of cooling performance in both single-cell and battery-module applications. Specifically, the CPCM (P1) prepared using large-pore-size (low PPI, such as 10 PPI) copper foam provides the best heat dissipation and cooling effect, followed by medium pore size (20 PPI, P2), while small pore size (high PPI, 30 PPI, P3) results in the least effective performance.

#### 3.1.5. Anti-Leakage Performance

**Figure 7:**
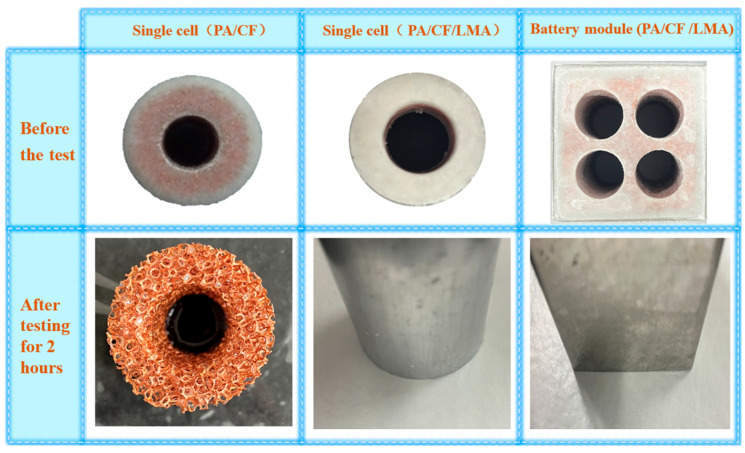
Images of the various samples before and after undergoing continuous heating at 65 °C.

Figure 7 visually compares the morphological changes and leakage conditions of PA/CF CPCM and PA/CF/LMA CPCM encapsulated with LMA after being heated at 65 °C for various durations. After being heated for two hours, the CPCM made from copper foam/paraffin exhibited nearly complete paraffin leakage, with the copper foam framework structure almost fully exposed and only a small amount of paraffin residue remaining. This occurred because the surface of the copper foam used in the experiment did not provide sufficient adhesion during the melting of the paraffin. As a result, the liquid paraffin was unable to adhere firmly to the copper foam framework, leading to progressive leakage during the melting process until complete leakage occurred. The leakage rate was close to 100%. In contrast, the LMA-encapsulated PA/CF/LMA CPCM retained its original shape after being heated at 65 °C for 2 h, with no visible traces of liquid paraffin leakage on the filter paper. This is because the selected LMA has a melting point higher than 65 °C and remains solid at this temperature, effectively serving as a physical encapsulation barrier. This result clearly confirms that LMA encapsulation significantly enhances the thermal stability and anti-leakage performance of PA/CF CPCM.

### 3.2. Thermal Management Performance and Analysis of CPCM

#### 3.2.1. Single Cell

**Figure 8:**
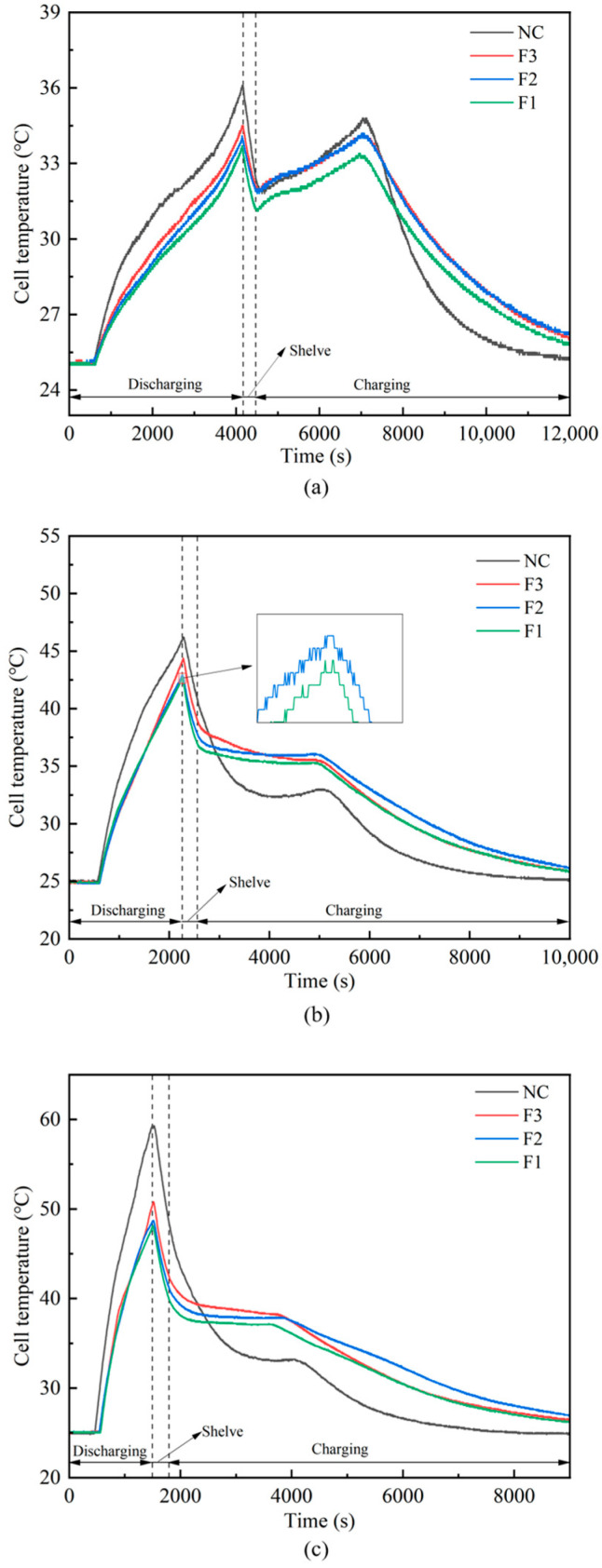
Temperature curve of single cell under (**a**) 1C; (**b**) 2C; (**c**) 3C. Notes: “NC”: natural convection, “F1–F3”: LMA-encapsulated PA/CF CPCMs with 10/20/30 PPI copper foam.

**Figure 9:**
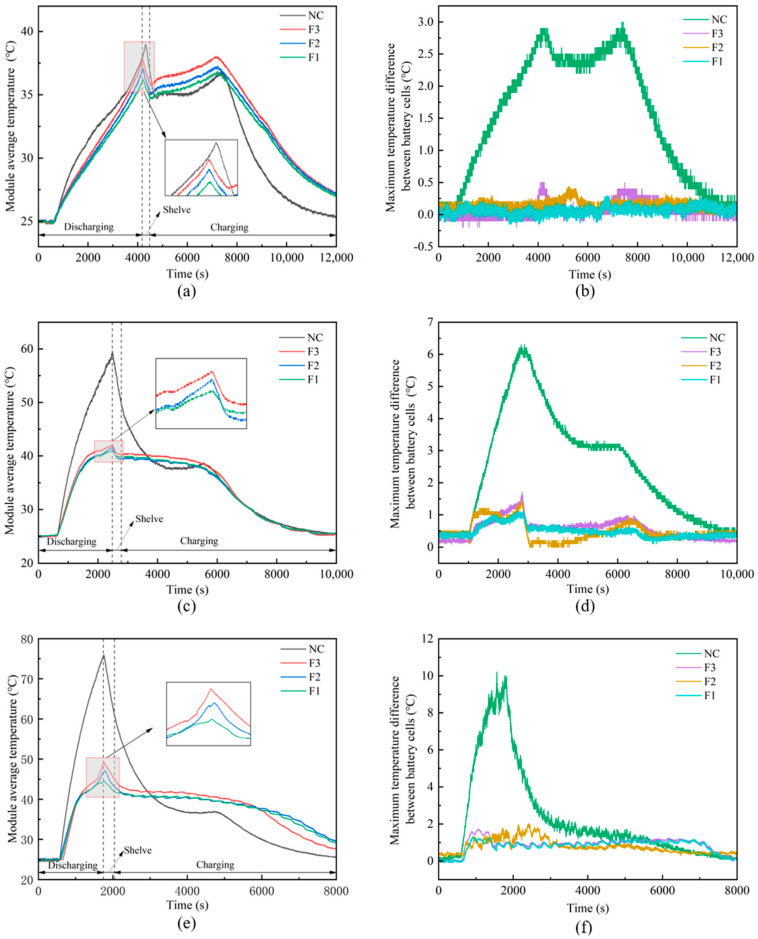
Temperature fluctuation of the battery module and maximum temperature discrepancy among cells in the module under (**a**,**b**) 1C; (**c**,**d**) 2C; (**e**,**f**) 3C. Notes: “NC”: natural convection, “F1–F3”: LMA-encapsulated PA/CF CPCMs with 10/20/30 PPI copper foam.

To evaluate the thermal regulatory performance of various materials for single lithium-ion battery cells, Figure 8 presents the temperature variation curves at the center of the battery surface under natural convective cooling conditions (NC) as well as when using three types of PA/CF/LMA CPCM with varying pore sizes (all encapsulated by LMA) (F1: 10 PPI, F2: 20 PPI, F3: 30 PPI) for cycles with 1C charging followed by discharge rates of 1C, 2C, and 3C. Under the constant current scheme, as the discharge rate increases (from 1C to 3C), the discharge duration in Figure 8 and Figure 9 is significantly reduced. On the contrary, the subsequent charging time remained consistent throughout all tests because a constant 1C charging rate was used. Under 1C discharge (Figure 8a), the maximum operating temperature of the battery under natural convection conditions reaches 36.1 °C, while the peak temperature with F3, F2, and F1 CPCM are 34.3 °C, 33.9 °C, and 33.5 °C, respectively. Under 2C discharge (Figure 8b), the battery peak temperature reaches 46.2 °C under natural convection, while the peak temperature with F3, F2, and F1 CPCM is reduced to 44.3 °C, 42.9 °C, and 42.7 °C, respectively. Under 3C discharge (Figure 8c), the battery peak temperature reaches 59.3 °C under natural convection, whereas with F3, F2, and F1 CPCM, the peak temperature is lowered to 50.3 °C, 48.5 °C, and 47.9 °C, respectively. A comprehensive analysis of the three operating conditions shows that, compared to natural convection, all CPCM configurations effectively reduce the peak battery temperature. As the PPI of the copper foam decreases, the efficiency of thermal transfer from the battery to the external surroundings increases. Specifically, F1 (10 PPI) demonstrates the best performance, followed by F2 (20 PPI), while F3 (30 PPI) is the least effective. This pattern aligns with the findings from heat conductivity tests as well as infrared analysis. When encapsulated composite phase change materials are utilized, they operate by serving as a thermal buffer—storing thermal energy during heating phases and discharging it during cooling phases. The superior performance of CPCM results from the synergistic effects of three factors: (a) the elevated thermal conductivity exhibited by LMA; (b) the superior thermal conduction property of the copper foam skeleton and its ability to direct heat flow; (c) the absorption of significant quantities of latent heat in the course of the phase change in paraffin. Among these, high thermal conductivity—especially that provided by low-PPI copper foam—is crucial for the efficient absorption and transfer of heat from the battery by CPCM.

#### 3.2.2. Battery Module

To further evaluate the effectiveness of the fabricated CPCM in thermal management, it was integrated into a battery module to assess its cooling performance. Figure 9a,c,e display the temperature variation curves of the battery module under natural convection conditions (NC) and with the application of three PA/CF/LMA CPCM types (F1: 10 PPI, F2: 20 PPI, F3: 30 PPI), at discharge rates of 1C, 2C, and 3C (with all charging conducted at 1C). The findings also show that CPCM delivers stable cooling performance for both individual cells and battery modules. Among the tested materials, the use of F1 showed the most pronounced cooling effect. When F1 was employed, the maximum temperature ( $T_{max}$ ) measured under 1C, 2C, and 3C discharge rates were reduced to 35.5 °C, 41.0 °C, and 44.6 °C, respectively. In contrast to natural convection cooling, the achieved maximum temperature drops were 3.4 °C, 18.6 °C, and 31.3 °C, respectively. The observed battery temperature attenuation is primarily attributed to the heat absorption by LMA and paraffin during battery operation. This is pivotal for lithium-ion battery thermal management, particularly during high-rate charge–discharge processes. The elevated thermal conductivity facilitates efficient heat extraction from the battery, mitigating overheating risks and improving the system’s heat dissipation efficiency. Figure 9b,d,f depict the maximum temperature difference among the battery cells within the module under natural convection (NC) conditions and for three types of PA/CF/LMA CPCMs (F1: 10 PPI, F2: 20 PPI, F3: 30 PPI) at discharge rates of 1C, 2C, and 3C, under different operating conditions. Under natural convection conditions, the maximum temperature differences ( ${\Delta T}_{max}$ ) at discharge rates of 1C, 2C, and 3C reached 2.9 °C, 6.3 °C, and 10.2 °C, respectively, exceeding the safety threshold. After applying F1, the maximum temperature difference in the lithium-ion battery is reduced to 0.3 °C at a discharge rate of 1C. The 2C discharge rate is reduced to 1.1 °C. At the 3C rate, it is reduced to 1.2 °C. After adopting CPCM, the maximum temperature difference between battery cells within the battery module is maintained within 2 °C. This improvement is mainly attributed to the presence of a highly conductive copper foam, which rapidly conveys the battery’s heat to the CPCM, which subsequently stores the thermal energy through its phase change, ensuring the secure operation of the battery module. It is observable that employing LMA-encapsulated CF/PA CPCM contributes to a more homogeneous temperature distribution within the battery module. The incorporation of CPCM not only helps lower the battery temperature but also significantly reduces the internal temperature difference in the battery module. This ensures that temperatures are maintained within safe operating limits, thereby improving safety throughout the cycling process. As shown in Table 3, a comparative analysis was conducted with the recent research on CPCM, providing a background for the performance of our material system. Data shows that the CPCM (F1) we studied achieved a competitive maximum temperature (44.6 °C) and minimum temperature difference (1.2 °C) at a 3C discharge rate, highlighting its outstanding capabilities in lithium battery thermal management. This work provides a fundamental understanding of thermal management for high-performance batteries and promising material solutions. Future work will expand the investigation to include a wider range of foam architectures and operational temperature ranges. Meanwhile, the integration of a denser thermal sensor network is planned to construct a detailed three-dimensional thermal model that can inform the rational, predictive design of larger-scale battery systems.

## 4. Conclusions

This study successfully fabricated a CPCM with significantly improved thermal conductivity, utilizing LMA-encapsulated CF/PA to target the thermal management needs of lithium-ion batteries. The key thermophysical properties of CPCM were evaluated, and the effects of copper foam pore size and different types of CPCM on the thermal management performance of both individual cells and battery modules were investigated. The key conclusions are summarized below.

Analysis of the impregnation effect indicates that the impregnation ratio of paraffin into copper foam decreases as PPI increases. The dense and irregular pore structure of high-PPI (small pore size) copper foam hinders complete paraffin infiltration.

Thermal conductivity tests and infrared thermal imaging analysis reveal that CPCM’s thermal conduction capability drops as copper foam PPI values rise. The PA/CF/LMA CPCM (F1) prepared using 10 PPI copper foam demonstrates the highest heat conduction efficiency (4.42 W·m^−1^·K^−1^) and the most effective heat dissipation and cooling effect.

In lithium-ion battery thermal regulation scenarios, the F1 CPCM shows superior comprehensive temperature control capabilities. Under 3C high-rate discharge conditions, the maximum temperature of the battery module is significantly reduced from 75.9 °C under natural convection to 44.6 °C, a decrease of 31.3 °C. More importantly, the maximum temperature difference between individual cells within the module is dramatically lowered from 10.2 °C under natural convection to just 1.2 °C. This leads to a more uniform temperature distribution and substantially safer module operation.

## Figures and Tables

**Table 1:** Experimental conditions.

Step	Condition (Cell)	Condition (Battery Module)
Rest	10 min	10 min
Constant Current (CC) Discharge	Cut-off: 2.75 V Current: 3.2 A (1C), 6.4 A (2C), 9.6 A (3C)	Cut-off: 5.0 V Current: 6.4 A (1C), 12.8 A (2C), 19.2 A (3C)
Rest	5 min	5 min
Charge (CC-CV)	4.2 V, 3.2 A	8.4 V, 6.4 A
Total Cycles	2	2

**Table 2:** Parameters of CF/PA composite CPCMs.

Copper Foam Pore Size	*m*_before_ (g)	*m*_after_ (g)	$\rho_{CPCM}$ (g/cm^3^)	*ε*	*m*_theoretical_ (g)	*m*_actual_ (g)	*η* (%)
10 PPI	5.873	24.322	0.367	0.935	18.7	18.449	98.658
20 PPI	4.674	21.493	0.229	0.955	19.1	17.891	93.670
30 PPI	3.446	20.571	0.215	0.965	19.3	17.125	88.731

**Table 3:** Benchmarking of thermal performance: this work versus recent composite phase change material system studies.

CPCM System	*T*_max_ (°C)	Δ*T*_max_ (°C)	Discharge Rate	Reference
polyethylene glycol, expanded graphite/ammonium polyphosphate (APP)/MXene/Zinc hydroxy stannate (ZHS)	57.03	5	3C	[46]
Polyethylene glycol (PEG)/EG/Diphenylmethane diisocyanate (MDI)/Melamine (MA)/9,10-dihydro-9-oxa-10-phospha-phenanthrene-10-oxide (DOPO) (PMDM)	55	5.5	2C	[47]
polyethylene glycol (PEG)/hexamethylene diisocyanate (HDI)/EG/hexagonal boron nitride (H-BN)/carbon nanotubes (CNTs)/silicon carbide (SiC)	50.2	3	3C	[30]
KAl(SO_4_)_2_·12H_2_O hydrated salts/Na_2_SO_4_·10H_2_O/EG/open-cell polyurethane foam	52.47	2.2	1.5C	[48]
PA/EG/bacterial cellulose (BC)	47	4	3C	[49]
PA/CF/LMA (This work)	44.6	1.2	3C	\

## Data Availability

The original contributions presented in this study are included in the article. Further inquiries can be directed to the corresponding authors.
